# Nature Notes

**Published:** 1913-05-10

**Authors:** 


### NATURE NOTES.

The Arrival of the Summer Birds—The First Swallow—Hatching Prospects—A Robin's Nest—The Wren's Nest—Traditions and Peculiarities.

The blackthorn winter has been keen, but now that the thorn is in flower it will pass. Why are the most of us surprised annually by the sharp spell of biting wind that comes with the first week of April—a time that the rustic anticipates and calls the blackthorn winter? It is the one regular feature of England's weather, as regular in its coming as the migrant birds.

I do not suppose the summer birds are later this year than usual. My observations make me believe that their time of arrival varies little. This year I have not seen any kind as early as I expected, but I doubt if the English weather makes any difference to the migrations to England. Possibly the weather of other lands decides the period. Perhaps the assumption of breeding plumage is the impulse that starts the northward flight. This year, though, I have seen the migrants late. Almost to the day the chiff-chaff is first heard on March 23. I have not heard it yet, though on Easter Sunday I thought I saw one slip along a thick hedge. I saw the sand-martins on April 20, and on the 23rd I saw swarms of them round a cliff of sand in which they nest. The first swallow I saw was in the Kennet Valley on April 21. I heard the cuckoo on the same day.

Nor do I think that the birds that stay with us throughout the year are earlier than usual at nesting, although the winter has been mild. I will except the rooks; I do think they were sitting earlier than in most years. On April 21 I saw a young missel-thrush fledged and flying that had already left the nest. The missel-thrush is an early nester, but I do not remember finding its eggs in March. Unfortunately I have few notes. Though keen to observe, I am too lazy to record. My garden, orchards, and paddocks are a bird sanctuary. Last year I found over a hundred nests, chiefly of the commoner sorts. This year the promise is good. Several blackbirds and thrushes have hatched already. Others are nesting. In a thick thorn hedge a blackbird is sitting, and a hedge-sparrow but a yard away has to-day, April 25, laid her second egg. Three yards off in some privet a thrush is rearing four nestlings that are nearly ready to fly. A robin has a wonderful nest in the side of the haystack. The material of the outside is dry bracken from a stack of bracken used for horse litter. The nest is cunningly contrived behind the down-hanging hay so that it is scarcely noticeable. At first sight I took it for the domed nest of a wren. The bird when on the nest is completely hidden. She slips in and out through a scarcely perceptible hole in the hay. I hope she may have luck this year. Last year she nested on the ground in a tussock beneath an apple tree, and hatched. Unfortunately, some ducks foraging across the orchard came upon the brood and devoured it. Ducks and chickens are very destructive to ground-nesting birds. They also catch and kill mice. I was much amused a fortnight ago with watching the agility with which certain long-legged hens in a neighbour's rick-yard ran down and caught the mice that fled from a wheat stack that was being threshed.

Two days ago I saw a wren carrying nesting material near by my pig-sty. I have not yet found the nest. I am looking for it because I am especially interested in wrens' nests. As a boy I believed that if I touched a wren's nest, or even too curiously inspected it, the bird probably would desert it. Yet I knew that when there were eggs in the nest one might take one or two and the bird would sit on and hatch the rest. There was a tradition we believed that if one found a wren's nest with two eggs in it and took one and next day took another, and next day another, and so on for days, that the bird would lay an egg a day for ninety-nine days and then a hundredth egg, a black one, and die on the nest. To our honour be it said we decided the experiment too cruel to be tried. Though we boys found we could take eggs from a wren's nest without driving the bird to desert, we also observed that often when we found a wren's nest completed but still eggless—a fact that could be only ascertained by inserting a finger—on visiting the nest again after an interval it was usually eggless and apparently deserted. We believed that it always was deserted, and deserted because the bird was jealous of its nest being touched. Usually we then pulled it out. Sometimes also on a later visit we found partly-built nests that had got no further. These also we usually pulled out. We called them cocks' nests, and believed, from tradition I suppose, that these were rough temporary shelters made by cock wrens to shelter them on extra cold nights. Age with its less ready faith brought me to discard belief in the black hundredth egg and in many of the other ninety-nine that should precede it, but still I had faith in the cocks' nests to a modified extent. I thought that wrens rather frequently began a nest and for reasons or from caprice abandoned it and the site. Blackbirds often will commence to build a nest, suddenly desist, and begin and complete another nest within a foot or two of the first. I believed also that the wren would desert her nest more readily than do other birds if it were disturbed.

But last year a wren that dwelt in my garden gave me reason to reconsider my ideas. By a path near a poplar tree is a pink May tree overgrown with honeysuckle. About four feet from the ground a wren began a nest between the honeysuckle stems that encircled the trunk of the May tree. For a day or two I often saw her collecting moss from the poplar tree and taking it to the nest. Suddenly she left her work. It remained for days a typical "cock's nest." I thought she had resented my watchings and deserted the site. But quite a fortnight later I observed that the nest had grown larger, and I saw the bird building day after day till the nest seemed complete. For some days I did not touch the nest lest I should cause the bird to desert, but not seeing her at the nest and being curious to know if she had eggs, I ventured to put a finger in. The nest was complete, but had no eggs in it.

For nearly a month the nest remained empty. I did not see the bird visit it. Some days it was soaked by rain. Usually I pull out all old nests and deserted nests in my garden, but this nest was pretty and of interest to visitors, so I left it. One day I was surprised to see the wren come out of it again. I put in a finger, and felt eggs. Eventually the bird finished her clutch and successfully hatched it This is the point of my story. If I had pulled out the "cock's nest" I should not have seen it completed, and should have thought it an abandoned attempt. If I had pulled out the completed nest after I had concluded that the bird had deserted it I should have believed she had finally deserted it. But because, almost by accident, I left it, I saw a "cock's nest" after an interval become a completed nest, and that apparently a deserted nest, and then this apparently deserted nest again in use and successfully used as a nursery from whence issued a happy family. This year I am watching to find out if it is customary for wrens to start to build long before the nest is required and to work at the nest at intervals rather than continuously, and to leave the nest ready for eggs long before the time comes to deposit them. Should this appear in print before nesting time is over, if any who reads feels interest in and finds opportunity for watching the nesting of the wrens I should be glad to learn what he may observe.

